# The Efficacy and Safety of Telerehabilitation for Fibromyalgia: Systematic Review and Meta-analysis of Randomized Controlled Trials

**DOI:** 10.2196/42090

**Published:** 2023-04-25

**Authors:** Yong-Qiang Wu, Yi Long, Wei-Jie Peng, Cheng Gong, Yue-Quan Liu, Xu-Miao Peng, Yan-Biao Zhong, Yun Luo, Mao-Yuan Wang

**Affiliations:** 1 Gannan Medical University GanZhou China; 2 Department of Rehabilitation Medicine The First Affiliated Hospital of Gannan Medical University GanZhou China

**Keywords:** telerehabilitation, fibromyalgia, systematic review, meta-analysis, rehabilitation, chronic pain, pain, musculoskeletal, monitoring, intervention, consultation, education, efficacy, safety

## Abstract

**Background:**

Fibromyalgia is a chronic pain syndrome characterized by persistent and widespread musculoskeletal pain. Telerehabilitation is a promising treatment for patients with fibromyalgia through long-term monitoring, intervention, supervision, consultation, and education.

**Objective:**

This study aimed to perform a comprehensive systematic review and meta-analysis of the efficacy and safety of telerehabilitation in patients with fibromyalgia.

**Methods:**

Randomized controlled trials (RCTs) related to fibromyalgia and telerehabilitation were systematically searched in the PubMed, PEDro, Cochrane Library, ScienceDirect, Ovid MEDLINE, Embase, and Web of Science databases from inception to November 13, 2022. Two independent researchers screened the literatures and evaluated the methodological quality using the Cochrane Risk of Bias Tool. The outcome measures included the Fibromyalgia Impact Questionnaire scale, pain intensity, depression, pain catastrophizing, quality of life (QoL), and adverse events. Pooled effect sizes were calculated by Stata SE 15.1; a fixed effects model was used when *I*^2^<50%, whereas a random effects model was used when *I*^2^≥50%.

**Results:**

A total of 14 RCTs with 1242 participants were included in this meta-analysis. The pooled results indicated that the telerehabilitation improved the Fibromyalgia Impact Questionnaire score (weighted mean difference –8.32, 95% CI –11.72 to –4.91; *P*<.001), pain intensity (standardized mean difference [SMD] –0.62, 95% CI –0.76 to –0.47; *P*<.001), depression levels (SMD –0.42, 95% CI –0.62 to –0.22; *P*<.001), pain catastrophizing (weighted mean difference –5.81, 95% CI –9.40 to –2.23; *P*=.001), and QoL (SMD 0.32, 95% CI 0.18 to 0.47; *P*<.001) in patients with fibromyalgia compared to control interventions. Only 1 RCT reported a mild adverse event of telerehabilitation; the other 13 RCTs did not mention this.

**Conclusions:**

Telerehabilitation can improve the symptoms and QoL of fibromyalgia. However, the safety of telerehabilitation remains uncertain due to the lack of sufficient evidence for the management of fibromyalgia. More rigorously designed trials are needed in the future to verify the safety and efficacy of telerehabilitation in fibromyalgia.

**Trial Registration:**

PROSPERO CRD42022338200; https://tinyurl.com/322keukv

## Introduction

### Background

Fibromyalgia is a common chronic pain syndrome characterized by persistent and widespread musculoskeletal pain that is usually accompanied by sleep disruption, fatigue, cognitive dysfunctions, and depressed mood [1,2]. The persistent physical and psychological symptoms of fibromyalgia lead to a decrease in patients’ quality of life (QoL) and cause a huge economic burden on their families and society [3,4]. The main goals of fibromyalgia treatment are to reduce symptoms, maintain function, and improve QoL. Various treatments have been recommended for the management of fibromyalgia, such as medication, exercise, education, and psychotherapy [5-7]. However, patients with fibromyalgia require long-term clinical management and follow-up [8].

Telerehabilitation is defined as “the provision of rehabilitation services through information and telecommunications technology” [9,10], which can provide the assessment, intervention, supervision, education, monitoring, consultation, and prevention of disease [11]. Telerehabilitation can effectively facilitate real-time communication between patients and health care providers and increase patients’ access to medical help from specialized physicians and therapists [12,13]. Telerehabilitation also can tailor long-term and ongoing telerehabilitation services according to the needs of the patients [14]. Telerehabilitation has been widely used in the management of neurological [14], cardiorespiratory [15], and musculoskeletal disorder [16]. In particular, telerehabilitation has been recommended for managing chronic pain worldwide during the current COVID-19 pandemic situation [17].

With the rapid development of telecommunication technology, a number of randomized controlled trials (RCTs) on the application of telerehabilitation in patients with fibromyalgia have been published recently. For example, Hernando-Garijo et al [18] suggested that video-guided, aerobic exercise–based telerehabilitation could effectively reduce pain and improve psychological distress in patients with fibromyalgia. Lee et al [19] made remote real-time pain monitoring and rehabilitation interventions for fibromyalgia using wearable device. In addition, Molinari et al [20] and Yuan et al [21] found a positive effect of a mobile app or multimedia interactive system–based telerehabilitation intervention on individuals with fibromyalgia. However, few systematic reviews have summarized the characteristics, efficacy, and safety of telerehabilitation in fibromyalgia. According to our knowledge, only one review by Bernardy et al [22] has investigated the effects of internet-delivered psychotherapy in individuals with fibromyalgia. However, this study only focused on internet-delivered psychotherapy and ignored other types of telerehabilitation services, such as mobile apps, videoconferencing, web-based education, and wearable devices.

### Objectives

Therefore, this study aimed to perform a comprehensive systematic review and meta-analysis of the efficacy and safety of telerehabilitation in patients with fibromyalgia.

## Methods

This study was carried out by adhering to the PRISMA (Preferred Reporting Items for Systematic Reviews and Meta-Analyses) guidelines and was registered in the International Prospective Register of Systematic Reviews (PROSPERO; CRD42022338200).

### Search Strategy

The PubMed, PEDro, Web of Science, ScienceDirect, Ovid MEDLINE, Cochrane Library, and Embase electronic databases were searched from inception to November 13, 2022. The following keywords and their combinations were used for the literature search: “Telerehabilitation,” “Telemedicine,” “eHealth,” “Videoconferencing,” “Wireless Technology,” “Mobile Health,” “Computer Communication Networks,” “Internet-Based Intervention,” “Fibromyalgia Syndrome,” and “Fibromyalgia.” The detailed search strategy is described in Multimedia Appendix 1. Furthermore, we manually searched the reference lists of eligible studies to identify additional studies.

### Study Selection Criteria

Two independent researchers (YQW and Y Long) selected studies following the framework of Population, Intervention, Comparison, Outcomes, and Study Design. Any divergence during the screening process was resolved through consultation between the 2 researchers. If there was still no agreement, a third independent researcher (WJP) decided after group discussion.

All potentially eligible studies were screened following the pre-established criteria. The inclusion criteria were as follows: (1) RCTs; (2) patients were adults aged ≥18 years with a diagnosis of fibromyalgia according to the American College of Rheumatology criteria; (3) the intervention of the experiment group was telerehabilitation, with no restrictions on the specific modality of technology and the content of telerehabilitation; and (4) the control group received non-telerehabilitation including usual care, waiting list, no intervention, or conventional face-to-face treatment. The exclusion criteria were as follows: (1) the study was a case report, conference abstract, or review article; (2) the study was not published in English; and (3) the study only compared different types of telerehabilitation.

### Data Extraction

Two investigators (YQW and XMP) independently completed the data extraction. The data included the first author, the year of publication, sample size, study design, characteristics of the participants (gender, age, diagnosis, duration of symptoms, etc), interventions, and outcome measures. When data were lacking, we tried to contact the corresponding author to obtain missing data. If there was a divergence between the reviewers, it was resolved by consultation with a third independent researcher (Y Luo).

### Methodological Quality Assessment

Two independent reviewers (CG and YQL) assessed the methodological quality using the Cochrane Risk of Bias Tool [23]. The tool includes the following domains: (1) sequence generation (selection bias), (2) allocation concealment (selection bias), (3) blinding of participants and personal (performance bias), (4) blinding of outcome assessment (detection bias), (5) incomplete outcome data (attrition data), (6) selective reporting (reporting bias), and (7) other sources of bias.

### Outcome Measures

Efficacy outcomes were evaluated using the Fibromyalgia Impact Questionnaire (FIQ) scale; pain intensity (eg, the Visual Analog Scale [VAS] and Brief Pain Inventory [BPI]); depression (eg, the Patient Health Questionnaire, the Beck Depression Inventory, the Center for Epidemiological Studies Depression Scale, and the Hospital Anxiety and Depression Scale-Depression); pain catastrophizing (Pain Catastrophizing Scale [PCS]); and QoL (Short Form Health Survey and the EQ-5D). Safety outcomes were evaluated based on adverse events.

### Statistical Analysis

The statistical analysis was performed using the Stata software (version 15.1 SE; StataCorp). If outcomes were measured using different scales, we used the standardized mean difference (SMD) and 95% CI. Otherwise, when outcomes were measured using the same scales, we used the weighted mean difference (WMD) and 95% CI. Statistical heterogeneity was evaluated using the *I^2^* statistic [24]. *I*^2^ values >75% indicated high levels of heterogeneity, *I*^2^ values ≥50% indicated medium levels of heterogeneity, and *I*^2^ values >25% indicated low levels of heterogeneity. If *I*^2^≥50%, we used the random effects model. Otherwise, we used the fixed effects model for data analysis. For the results of ≥10 included studies, publication bias in the meta-analysis was detected by performing Egger regression test and Begg rank correlation test [25,26].

## Results

### Study Inclusion

**Figure 1:**
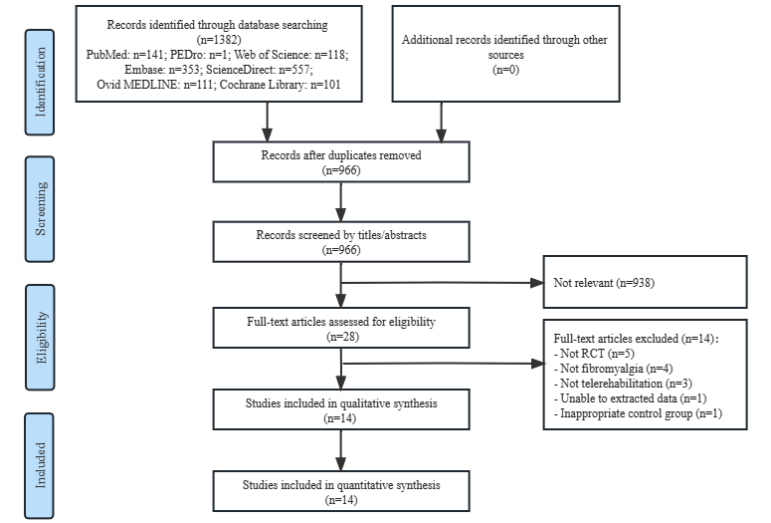
Flowchart of study selection. RCT: randomized controlled trial.

In total, 1382 records were identified from the 7 databases. After removing duplicates, screening the titles and abstracts, and reviewing the full-text, 1368 articles were excluded and 14 studies were considered eligible for this systematic review and meta-analysis [18-21,27-36] (Figure 1).

### Study Characteristics

Table 1 summarizes the main clinical characteristics of the included studies. The 14 RCTs included 1242 participants diagnosed with fibromyalgia. The mean age of the participants ranged from 39.7 to 55.5 years. The duration of the symptoms ranged from 6.21 to 20 years. The control intervention of the included studies involved a waiting list [30-32], usual care or treatment [18,19,27,28,33,35,36], pharmacologic therapy [29], daily activity [20], standard follow-up [34], and paper-based instruction [21]. The 14 RCTs reported different outcomes: 12 studies measured the FIQ scale [18-21,29-36], 8 assessed pain intensity [18,19,21,27,28,31,32,36], 11 assessed depression [18-20,27,28,30-33,35,36], 3 assessed pain catastrophizing [18,20,33], and 6 assessed QoL [19,28,31,34-36] (Table 1).

Table 2 summarizes the detailed protocols of telerehabilitation among the included studies. Regarding the medium of telerehabilitation, 8 studies used internet-based telerehabilitation [20,28,29,31-33,35,36], 5 used telephone- or mobile app–based telerehabilitation [18,21,27,30,34], and 1 used a wearable device to monitor and record real-time pain [19]. In terms of telerehabilitation programs, 6 studies used psychological interventions–based telerehabilitation [20,27,29,30,32,33], 2 used fibromyalgia self-management–based telerehabilitation [21,28], 3 used video-based guided exercise training telerehabilitation [18,35,36], 1 used a web-based pain course–based telerehabilitation [31], 1 provided real-time pain monitoring by a monitoring system [19], and 1 study provided web-based teleconsultation [34].

**Table 1:** Clinical characteristics of the included studies.

Study	Sample size, n	Sex (male/female), n	Age (years), mean (SD)	Duration of symptoms (years)	Interventions	Duration of intervention	Outcome index	Adverse event
Williams et al [28], 2010	EG^a^: 59 CG^b^: 59	EG: 3/56 CG: 3/56	EG: 50.17 (12.34) CG: 50.75 (10.58)	EG: 9.45 (6.85) CG: 9.34 (6.10)	EG: WEB-SM^c^ CG: UC^d^	6 months	BPI^e^, SF-36^f^, CES-D^g^, MFI^h^, MOS^i^, and STPI^j^	Not mention
Ang et al [27], 2010	EG: 15 CG: 13	EG: 0/17 CG: 0/15	EG: 50.5 (9.5) CG: 47.0 (12.4)	EG: 11.8 (4.6) CG: 12.3 (7.9)	EG: CBT^k^ CG: UC	6 weeks	NFR^l^ threshold, FIQ^m^-pain rating, and PHQ-8^n^	Not mention
Menga et al [29], 2014	EG: 17 CG: 27	Not reported	55	Not reported	EG: internet-based CBT CG: pharmacologic therapy	6 and 12 months	FIQ and Number of Tender Points	Not mention
Vallejo, et al [30], 2015	EG: 20 CG1: 20 CG2: 20	EG: 0/20 CG1: 0/20 CG2: 0/20	EG: 49.82 (11.01) CG1: 51.33 (10.03) CG2: 53.50 (8.56)	EG: 8.6 (7.85) CG1: 8.6 (7.23) CG2: 8.8 (6.94)	EG: iCBT^o^ CG1: waiting list CG2: CBT	10 weeks	FIQ, PCS^p^, HADS^q^, BDI^r^, and CPSS^s^	Not mention
Friesen, et al [31], 2017	EG: 30 CG: 30	EG: 2/28 CG: 1/29	EG: 49 (10) CG: 46 (13)	EG: 20 (9) CG: 13 (10)	EG: internet-based pain management CG: waiting list	8 weeks	FIQ-R^t^, BPI, GAD-7^u^, HADS, SF-12, and PSEQ^v^	Not mention
Hedman-Lagerlöf et al [32], 2018	EG: 70 CG: 70	EG: 2/68 CG: 1/69	EG: 51.8 (10.7) CG: 49.3 (10.0)	EG: 11.0 (7.3) CG: 9.2 (7.5)	EG: iExp^w^ CG: waitlist	10 weeks	FIQ, FIQ-pain, FFS^x^, WHO-DAS II^y^, depressive symptoms using the PHQ-9, GAD-7, and ISI^z^	Increased pain
Molinari et al [20], 2018	EG: 38 CG: 33	Not reported	51.08 (10.54)	13.1 (10.07)	EG: BPS^aa^ CG: daily activities	4 weeks	Depression BDI-II, PANAS^ab^, GSES-12^ac^, FIQ-R, and PCS	Not mention
Simister et al [33], 2018	EG: 33 CG: 34	3/64	39.7 (9.36)	10.16 (7.83)	EG: web-based ACT^ad^ + TAU^ae^ CG: TAU	2 months	FIQ-R, CES-D, FFMQ^af^, CFQ^ag^, and SF-MPQ^ah^	Not mention
Lee et al [19], 2019	EG: 14 CG: 11	EG: 0/14 CG: 0/11	EG: 42.8 (7.2) CG: 41.7 (11.2)	EG: 6.21 (10.5 CG: 7.13 (20.6)	EG: PAAS^ai^ CG: usual treatment	3 months	FIQ, VAS^aj^, BDI, and EQ-5D	Not mention
García-Perea et al [34], 2021	EG: 40 CG: 40	EG: 1/39 CG: 2/38	EG: 53.3 (7.49) CG: 55.5 (4.06)	Not reported	EG: web-based nursing consultation CG: standard follow-up	6 and 12 months	FIQ and SF-36	Not mention
Yuan et al [21], 2021	EG: 20 CG: 20	EG: 1/19 CG: 0/20	EG: 43.3 (8.4) CG: 42.1 (11.8)	EG: 8.9 (4.9) CG: 7.2 (5.8)	EG: ProFibro app CG: paper book	6 weeks	FIQ-R, VAS, WPI^ak^, symptom severity, and self-care	Not mention
Serrat et al [35], 2021	EG: 75 CG: 76	EG: 4/71 CG: 6/70	EG: 54.89 (8.94) CG: 53.82(8.45)	Not reported	EG: FIBROWALK + TAU CG: TAU	12 weeks	FIQ-R, TSK^al^, HADS-Anxiety and HADS-Depression, and SF-36	Not mention
Hernando-Garijo et al [18], 2021	EG: 14 CG: 14	EG: 0/14 CG: 0/14	EG: 51.81 (9.05) CG: 55.06 (8.51)	10.54 (7.4)	EG: telerehabilitation program CG: usual treatment	15 weeks	FIQ, VAS, Algometer score, PCS, HADS, 6-min Walk Test, and Arm Curl Test	Not mention
Serrat et al [36], 2022	EG1: 110 EG2: 110 CG: 110	EG1: 1/109 EG2: 3/107 CG: 7/103	EG1: 52.78 (8.64) EG2: 52.54 (9.78) CG: 53.48 (8.93)	15.6 (9.12)	EG1: FIBROWALK + TAU EG2: MPP^am^ + TAU CG: TAU	12 weeks	FIQ-R, VAS, HADS, and SF-36	Not mention

^a^EG: experimental group.

^b^CG: control group.

^c^WEB-SM: Web-Enhanced Behavioral Self-management.

^d^UC: usual care.

^e^BPI: Brief Pain Inventory.

^f^SF: Short Form Health Survey Questionnaire.

^g^CES-D: The Center for Epidemiological Studies Depression Scale.

^h^MFI: Multidimensional Fatigue Inventory.

^i^MOS: Medical Outcomes Study.

^j^STPI: State-Trait Personality Inventory.

^k^CBT: cognitive behavioral therapy.

^l^NFR: Nociceptive Flexion Reflex.

^m^PHQ: Patient Health Questionnaire.

^n^FIQ: Fibromyalgia Impact Questionnaire.

^o^iCBT: internet-delivered cognitive behavioral therapy.

^p^PCS: Pain Catastrophizing Scale.

^q^HADS: Hospital Anxiety and Depression Scale.

^r^BDI: Beck Depression Inventory.

^s^CPSS: Chinese version of the Perceived Stress Scale.

^t^FIQ-R: Revised Fibromyalgia Impact Questionnaire

^u^GAD-7: Generalized Anxiety Disorder 7-Item.

^v^PSEQ: Pain Self-Efficacy Questionnaire.

^w^iEXP: internet-delivered exposure treatment.

^x^FFS: Fatigue Severity Scale.

^y^WHO-DAS II: the World Health Organization Disability Assessment Schedule II.

^z^ISI: Insomnia Severity Index.

^aa^BPS: Best Possible Self.

^ab^PANAS: The Positive and Negative Affect Scale.

^ac^GSES-12: General Self Efficacy Scale.

^ad^ACT: acceptance and commitment therapy.

^ae^TAU: treatment as usual.

^af^FFMQ: The Five Facet Mindfulness Questionnaire.

^ag^CFQ: The Cognitive Fusion Questionnaire.

^ah^SF-MPQ: The McGill Pain Questionnaire-short form.

^ai^PAAS: Pain Assessment and Analysis System.

^aj^VAS: Visual Analog Scale for Pain.

^ak^WPI: Widespread Pain Index.

^al^TSK: The Tampa Scale for Kinesiophobia.

^am^MPP: Multicomponent Physiotherapy Program.

**Table 2:** Specific protocol of telerehabilitation among the included studies.

Study	Medium	Program
Williams et al [28], 2010	Internet	Web-Enhanced Behavioral Self-management program: (1) educational lectures; (2) education, behavioral, and cognitive skills; and (3) behavioral and cognitive skills designed to facilitate adaptive lifestyle changes for managing fibromyalgia
Ang et al [27], 2010	Telephone	CBT^a^ program: (1) time-contingent activity pacing; (2) pleasant activity scheduling; (3) relaxation; and (4) automatic thoughts and pain, cognitive restructuring, and stress management
Menga et al [29], 2014	Internet	CBT and interpersonal therapy: MoodGYM^b^ contains 5 modules based on cognitive reconstructing, relaxation, pleasant events, assertiveness training, and problem-solving
Vallejo et al [30], 2015	Mobile app	Internet-CBT: (1) psychoeducation about fibromyalgia and pain; (2) progressive relaxation training; (3) emotional training; (4) daily activities; and (5) cognitive restructuring and managing
Friesen et al [31], 2017	Internet	Pain Course: (1) web-based lessons (images and text presented in slide show format); (2) lesson summaries (images and text similar to a self-help book) combined with homework assignments; and (3) additional resources
Hedman-Lagerlöf et al [32], 2018	Internet	Internet-based exposure therapy: (1) psychoeducation; (2) refraining from avoidance behaviors; (3) approaching situations or behaviors normally avoided; and (4) relapse prevention program
Molinari et al [20], 2018	Internet	The Best Possible Self: (1) write down and imagine their best possible self; (2) patients could choose images, sounds, and videos from the Book of Life database; and (3) received SMS per week with reminders to practice their exercise and reinforcements
Simister et al [33], 2018	Internet	Web-based acceptance and commitment therapy: (1) acceptance; (2) values clarification; (3) medications, sleep hygiene, strategies for fibro fog and memory, exercise, and effective communication; (4) cognitive defusion (or “You are not your thoughts!”); and (5) mindfulness and self-as-context
Lee et al [19], 2019	Wearable device and mobile app	Real-time pain monitoring system: the PAAS^c^ device is reporting the severity of pain levels
García-Perea et al [34], 2021	Mobile app	Web-based nursing consultation: (1) training on how to use the system and (2) provided with properly documented information based on available scientific information relating to their disorder (fibromyalgia) and their regular medication schemes, with fact sheets on the suitability, secondary effects, contraindications, and interactions of such medication
Yuan et al [21], 2021	Mobile app	Self-care in fibromyalgia management: (1) patient education through animation; (2) self-monitoring with the FIQ^d^; (3) sleep strategies with guided imagery relaxation technique, stimulus control therapy, and sleep hygiene; (4) graded exercise program; (5) an eBook; and (6) hints
Serrat et al [35], 2021	Internet	FIBROWALK arm underwent a multicomponent strategy: (1) a link to a 60-min video (hosted on a private YouTube channel) was sent by email once a week for the following 11 weeks; (2) FIBROWALK virtual treatment (included PNE^e^, therapeutic physical exercise, self- management patient education, CBT techniques, and mindfulness training); and (3) homework exercises
Hernando-Garijo et al [18], 2021	Telephone	Telerehabilitation aerobic exercise program: (1) telerehabilitation sessions were based on low-impact rhythmic movements; (2) joint mobility exercises and active stretching; (3) the aerobic exercises were based on low-impact rhythmic movements guided by video; and (4) static stretching of the major muscles and breathing techniques
Serrat et al [36], 2022	Internet	The video-based FIBROWALK program: (1) a 60-min video; (2) FIBROWALK virtual treatment; and (3) homework exercises The video-based Multicomponent Physiotherapy Program: (1) 60-min video and (2) physiotherapy techniques (included PNE, therapeutic physical exercise, and self-management patient education)

^a^CBT: cognitive behavioral therapy.

^b^MoodGYM: a free interactive web-based program [37].

^c^PAAS: Pain Assessment and Analysis System.

^d^FIQ: Fibromyalgia Impact Questionnaire.

^e^PNE: Pain Neuroscience Education.

### Results From Quality Assessments

**Figure 2:**
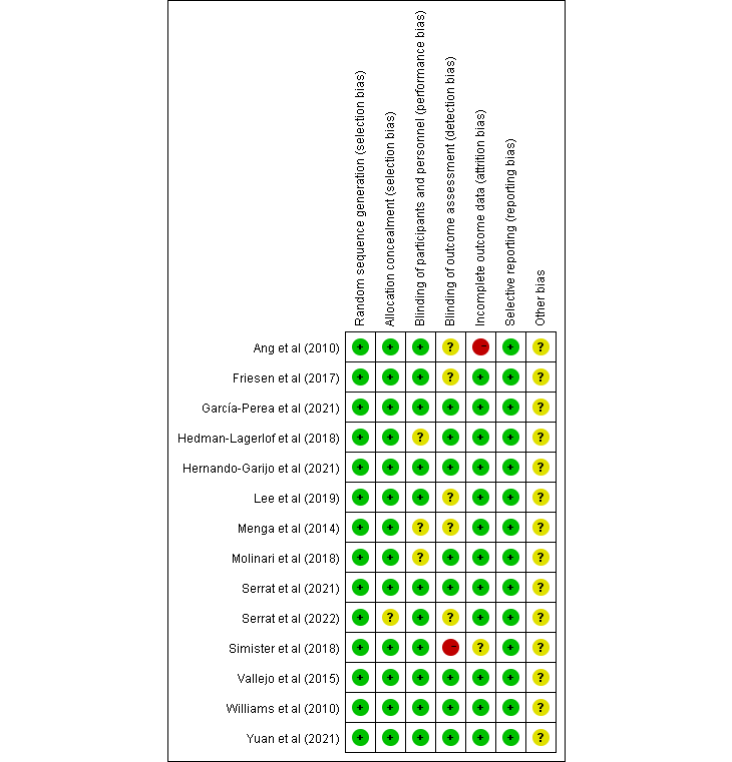
Risk of bias summary: review authors’ judgements about each risk of bias item for each included study [18-21,27-36].

**Figure 3:**
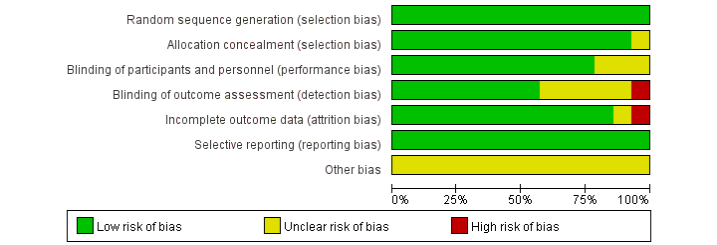
Risk of bias graph: review authors’ judgements about each risk of bias item presented as percentages across all included studies.

The risk of bias assessment according to the Cochrane criteria is shown in Figures 2 and 3. All trials reported a randomized method, providing a random sequence generation method, and one trial [36] reported an unclear allocation concealment method. One trial [33] was not blinded to the outcome assessment, and 5 trials [19,27,29,31,36] did not elaborate on whether the outcome assessment was blinded. Only three [20,29,32] of the 14 trials did not explicitly report whether they blinded the subjects. In addition, only 2 trials [27,33] mentioned dropout, but only 1 trial [27] did not elaborate on information processing.

### Effect of Telerehabilitation on the FIQ Scale

**Figure 4:**
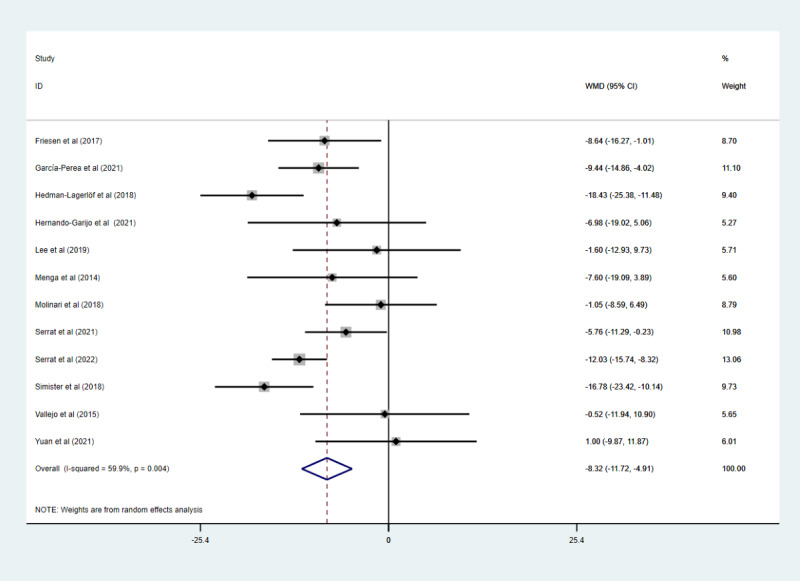
The effect of telerehabilitation on the Fibromyalgia Impact Questionnaire scale in fibromyalgia. WMD: weighted mean difference.

Twelve studies with 1076 participants [18-21,29-36] reported FIQ scale data. Owing to the existence of medium heterogeneity (*I*^2^=59.9%; *P*=.004), a random effects model was used. A meta-analysis showed that telerehabilitation had significantly reduced the FIQ scale score compared to the control group (WMD –8.32, 95% CI –11.72 to –4.91; *P*<.001; Figure 4).

### Effect of Telerehabilitation on Pain Intensity

**Figure 5:**
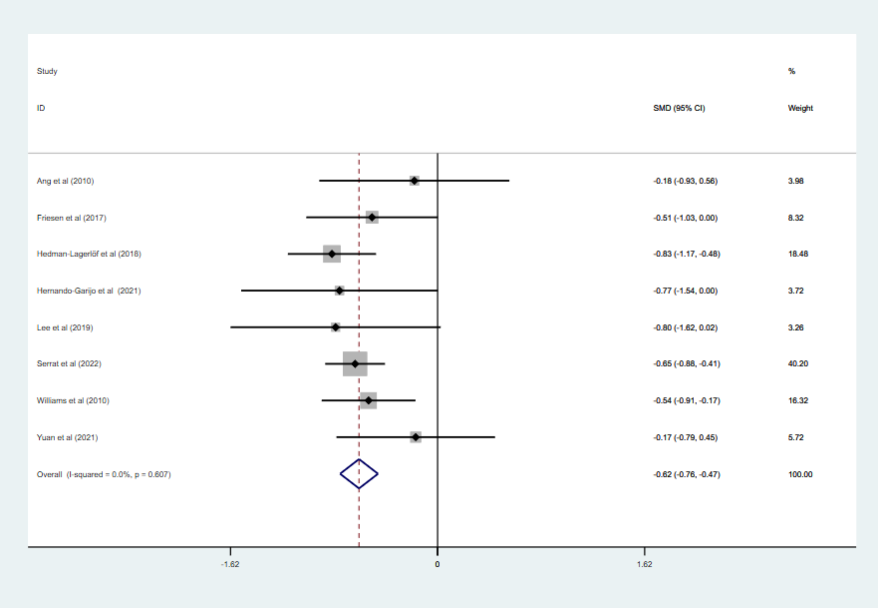
The effect of telerehabilitation on pain intensity in fibromyalgia. SMD: standardized mean difference.

Eight studies with 769 participants assessed pain intensity. Four studies used the VAS [18,19,21,36], 2 used FIQ-pain [27,32], and 2 used the BPI [28,31]. Due to the low heterogeneity (*I*^2^=0%; *P*=.61), a fixed effects model was used in the meta-analysis (Figure 5). The pooled results indicated that telerehabilitation had significantly improved pain intensity compared to the control group (SMD –0.62, 95% CI –0.76 to –0.47; *P*<.001).

### Effect of Telerehabilitation on Depression

**Figure 6:**
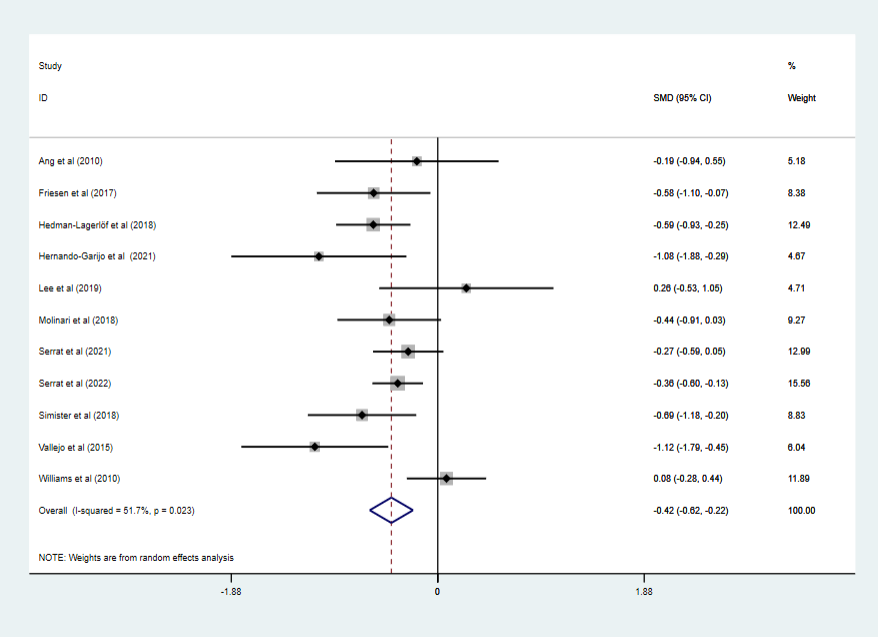
The effect of telerehabilitation on depression in fibromyalgia. SMD: standardized mean difference.

Eleven studies with 1058 participants assessed the depression levels. Two studies used the Patient Health Questionnaire [27,32], 2 used the Center for Epidemiological Studies Depression Scale [28,33], 3 used the Beck Depression Inventory [19,20,30], and 4 used the Hospital Anxiety and Depression Scale-Depression scale [18,31,35,36]. Due to the medium heterogeneity (*I*^2^=51.7%; *P*=.02), pooled results under a random effects model (Figure 6) indicated that telerehabilitation reduced the depression levels of patients with fibromyalgia (SMD –0.42, 95% CI –0.62 to –0.22; *P*<.001).

### Effect of Telerehabilitation on Pain Catastrophizing

**Figure 7:**
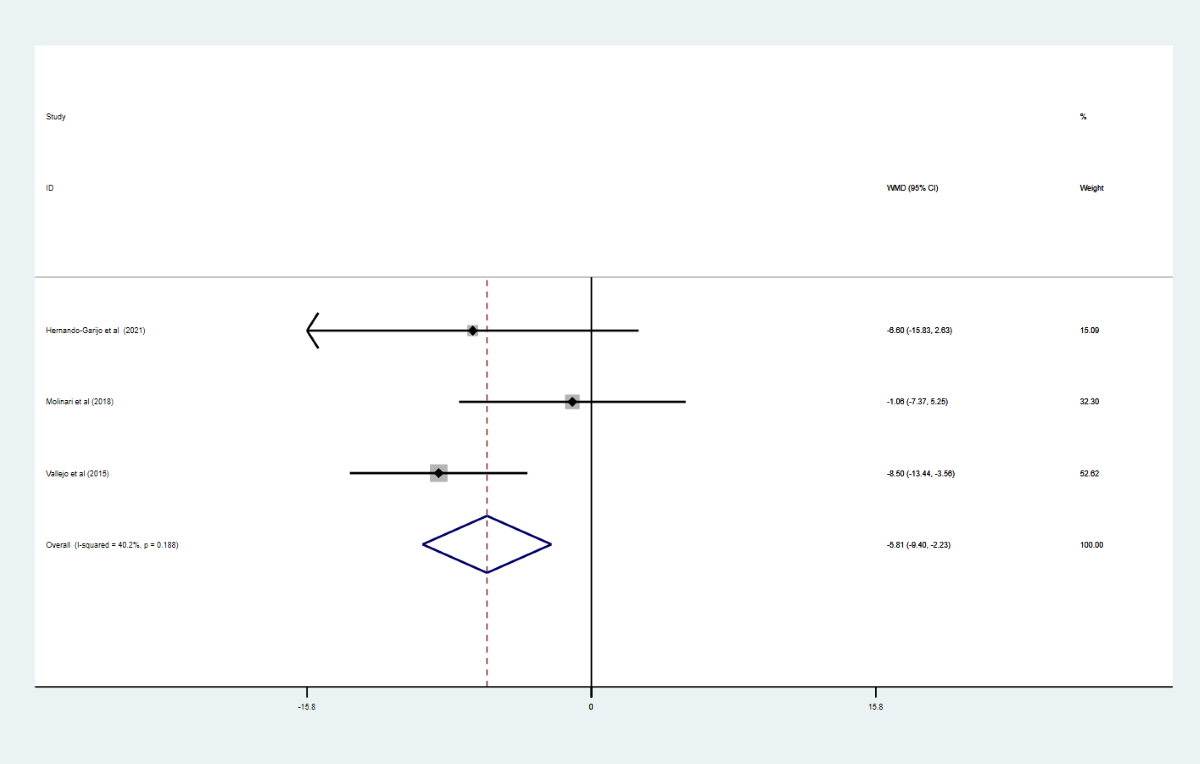
The effect of telerehabilitation on pain catastrophizing in fibromyalgia. WMD: weighted mean difference.

Pain catastrophizing was measured using the PCS. Three studies with 139 participants reported PCS data [18,20,30]. Because of the low heterogeneity (*I*^2^=40.2%; *P*=.19), a fixed effects model was used in the meta-analysis (Figure 7). The pooled results showed that telerehabilitation had significantly reduced PCS scores compared to the control group (WMD –5.81, 95% CI –9.40 to –2.23; *P*=.001).

### Effect of Telerehabilitation on QoL

**Figure 8:**
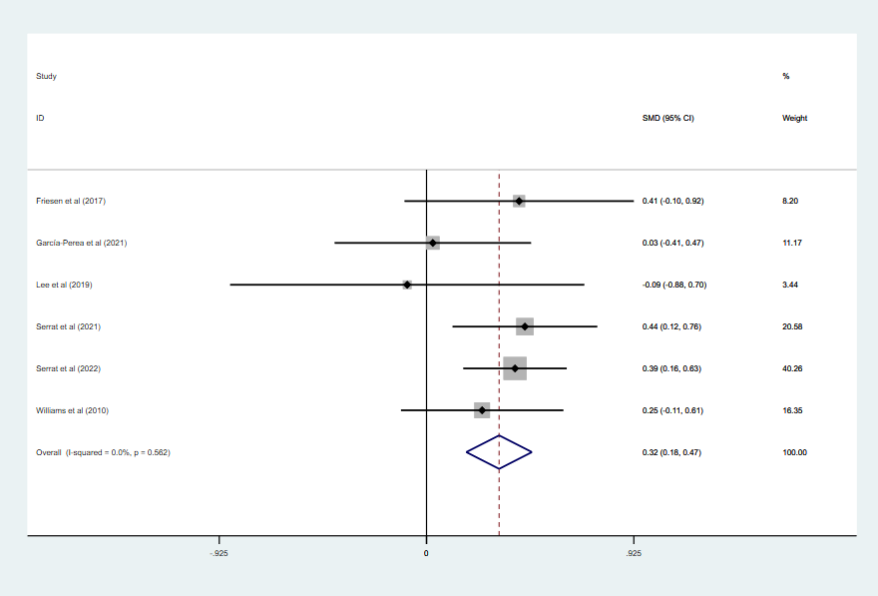
The effect of telerehabilitation on the quality of life in fibromyalgia. SMD: standardized mean difference.

Six studies with 764 participants assessed QoL. The QoL was assessed using the Short Form Health Survey in 5 studies [28,31,34-36] and the EQ-5D in 1 study [19]. In these studies, we found no significant heterogeneity in the QoL (*I*^2^=0%; *P*=.56). Pooled results under a fixed effects model (Figure 8) indicated that telerehabilitation had significantly improved the QoL compared with the control group (SMD 0.32, 95% CI 0.18 to 0.47; *P*<.001).

### Adverse Events

Only 1 RCT [32] reported an adverse event; the other 13 RCTs did not mention it. Hedman-Lagerlöf et al [32] reported that 34% of participants who received internet-based telerehabilitation experienced mild pain, but regression analysis showed no significant relationship between adverse events and telerehabilitation intervention.

### Publication Bias

No publication bias was found for the FIQ (Egger: *P*=.11; Begg: *P*=.30) and depression (Egger: *P*=.44; Begg: *P*=.44) in the funnel plot (Multimedia Appendix 2).

## Discussion

### Principal Findings

The aim of this meta-analysis was to summarize the current evidence about the efficacy and safety of telerehabilitation for patients with fibromyalgia. The results of this meta-analysis indicated that telerehabilitation can improve the FIQ score, pain intensity, depression, and QoL in patients with fibromyalgia compared to control interventions (eg, waiting list, pharmacologic therapy, and other active nonpharmacological therapies). Importantly, we also considered another important factor applied to patients with fibromyalgia: the safety of telerehabilitation. Only 1 study of the included studies reported mild adverse events after telerehabilitation [32]. Therefore, the evidence is not yet sufficient to draw firm conclusions about the safety of telerehabilitation for patients with fibromyalgia.

The key to whether telerehabilitation can be applied in patients with fibromyalgia is considering the clinical significance and the economic and social benefits. Our results indicate that telerehabilitation can effectively alleviate the symptoms in patients with fibromyalgia. Meanwhile, there are a lot of potential advantages of telerehabilitation, such as improving the efficiency of health professionals and providing people with various health care resources (eg, knowledge, fibromyalgia education, and exercise guidance) about fibromyalgia and personalized rehabilitation recommendations [38]. More importantly, telerehabilitation can overcome geographical barriers and provide medical care services to people in rural and remote communities and patients with fibromyalgia who cannot attend traditional face-to-face rehabilitation services, which is especially essential during the COVID-19 pandemic [39]. In addition, although none of the included studies in this meta-analysis reported cost-effectiveness, several previous studies have demonstrated that telerehabilitation can reduce the monetary and time costs of rehabilitation services [40,41].

### Comparison With Prior Work

In this meta-analysis, telerehabilitation was considered as a rehabilitation service of various telecommunication technology [42]. Currently, it is possible to apply telecommunication technology to rehabilitation service in clinical practice through cell phones, applications, virtual reality devices, and other electronic devices [43]. Telerehabilitation can deliver rehabilitation management services such as assessment, intervention, and patient consultation. The studies included in this meta-analysis primarily used the internet, telephone or mobile apps, and wearable devices to provide telerehabilitation services, including consultation, monitoring, education, and intervention. In addition, the conventional face-to-face management of fibromyalgia currently is mainly a nonpharmacological interventions tailored to individual symptoms, such as psychotherapy, exercise, and self-management strategies that involve active patient participation [44]. However, in this meta-analysis, the interventions of the included studies used 3 rehabilitation interventions, with psychological interventions being the mainstay, followed by video-guided exercise training and self-management.

Few systematic reviews have investigated the effects of telerehabilitation in patients with fibromyalgia. To the best of our knowledge, only 1 review by Bernardy et al [22] has investigated the effects of internet-delivered psychotherapy with fibromyalgia. They found that telerehabilitation reduced negative mood and improved QoL, which was consistent with our findings. However, in terms of pain intensity, there was no significant improvement in the telerehabilitation group compared to the control group, which is inconsistent with our results. This is because their study used an established dichotomous variable to assess pain, whereas our study used a continuous variable based on the VAS, BPI, and FIQ-pain to reflect pain improvement. Overall, this meta-analysis indicated that telerehabilitation has a positive effect on the FIQ score, pain intensity, depression, and QoL in patients with fibromyalgia.

### Challenges of Telerehabilitation for the Management of Fibromyalgia

Furthermore, there are several challenges to consider when telerehabilitation is applied in clinical settings. First, since telerehabilitation requires patients to be proficient in telecommunication technology, it may be difficult for patients unfamiliar with modern technology (eg, individuals with low IT literacy). In addition, some studies have suggested that the protection of patients’ privacy and supervision of relevant laws and regulations are also important factors influencing their willingness to use telerehabilitation [45,46]. Finally, the safety of telerehabilitation treatment remains unclear in patients with fibromyalgia. More research is required in the future to investigate the safety of telerehabilitation for patients with fibromyalgia.

### Limitations

This study had several limitations. First, the studies included in this meta-analysis were heterogeneous with respect to the medium and programs of telerehabilitation in the intervention group. Second, only 2 studies included a 6-month follow-up period, so this meta-analysis only examined the short-term effects of telerehabilitation. Therefore, the long-term effects are still unclear. Third, all eligible studies were published in English, and it is likely that relevant studies published in other languages have been omitted, resulting in a language bias.

### Clinical Implications

Our study found that telerehabilitation is an effective treatment approach for patients with fibromyalgia. Telerehabilitation can provide accessible and continuous rehabilitation medical services for patients with fibromyalgia who cannot attend traditional face-to-face services or are geographically remote, and it could enable patients to manage their disease at any time and place in a timely and appropriate manner.

More high-quality studies are required in the future to determine the efficacy of different forms of telerehabilitation and to focus on longer follow-ups to assess long-term outcomes in patients with fibromyalgia.

### Conclusion

Telerehabilitation can improve the FIQ score, pain intensity, depression level, pain catastrophizing, and QoL of patients with fibromyalgia. However, there was uncertainty about the safety of telerehabilitation, so rigorously designed trials are needed in the future to verify the safety and efficacy of telerehabilitation in fibromyalgia.

## Appendix

Multimedia Appendix 1 Search strategy.

Multimedia Appendix 2 Funnel plots.

Multimedia Appendix 3 PRISMA (Preferred Reporting Items for Systematic Reviews and Meta-Analyses) checklist.

## Abbreviations

BPI: Brief Pain Inventory
FIQ: Fibromyalgia Impact Questionnaire
PCS: Pain Catastrophizing Scale
PRISMA: Preferred Reporting Items for Systematic Reviews and Meta-Analyses PROSPERO: International Prospective Register of Systematic Reviews
QoL: quality of life
RCT: randomized controlled trial
SMD: standardized mean difference
VAS: Visual Analogue Scale
WMD: weighted mean difference

## References

[1] Giorgi V, Sirotti S, Romano ME, Marotto D, Ablin JN, Salaffi F, Sarzi-Puttini P (2022). Fibromyalgia: one year in review 2022. Clin Exp Rheumatol.

[2] Sarzi-Puttini P, Giorgi V, Marotto D, Atzeni F (2020). Fibromyalgia: an update on clinical characteristics, aetiopathogenesis and treatment. Nat Rev Rheumatol.

[3] Cabo-Meseguer A, Cerdá-Olmedo Germán, Trillo-Mata JL (2017). Fibromyalgia: Prevalence, epidemiologic profiles and economic costs. Med Clin (Barc).

[4] D'Onghia M, Ciaffi J, Ruscitti P, Cipriani P, Giacomelli R, Ablin JN, Ursini F (2022). The economic burden of fibromyalgia: a systematic literature review. Semin Arthritis Rheum.

[5] Kim SY, Busch AJ, Overend TJ, Schachter CL, van der Spuy I, Boden C, Góes Suelen M, Foulds HJ, Bidonde J (2019). Flexibility exercise training for adults with fibromyalgia. Cochrane Database Syst Rev.

[6] Cohen-Biton L, Buskila D, Nissanholtz-Gannot R (2022). Review of fibromyalgia (FM) syndrome treatments. Int J Environ Res Public Health.

[7] Kundakci B, Kaur J, Goh SL, Hall M, Doherty M, Zhang W, Abhishek A (2022). Efficacy of nonpharmacological interventions for individual features of fibromyalgia: a systematic review and meta-analysis of randomised controlled trials. Pain.

[8] Gota CE (2018). What you can do for your fibromyalgia patient. Cleve Clin J Med.

[9] Pastora-Bernal J, Estebanez-Pérez María-José, Molina-Torres G, García-López Francisco-José, Sobrino-Sánchez Raquel, Martín-Valero Rocío (2021). Telerehabilitation intervention in patients with COVID-19 after hospital discharge to improve functional capacity and quality of life. study protocol for a multicenter randomized clinical trial. Int J Environ Res Public Health.

[10] Duruturk N (2020). Telerehabilitation intervention for type 2 diabetes. World J Diabetes.

[11] Nizeyimana E, Joseph C, Plastow N, Dawood G, Louw QA (2022). A scoping review of feasibility, cost, access to rehabilitation services and implementation of telerehabilitation: implications for low- and middle-income countries. Digit Health.

[12] Rosen MJ (2004). Telerehabilitation. Telemed J E Health.

[13] Xie SH, Wang Q, Wang LQ, Wang L, Song KP, He CQ (2021). Effect of internet-based rehabilitation programs on improvement of pain and physical function in patients with knee osteoarthritis: systematic review and meta-analysis of randomized controlled trials. J Med Internet Res.

[14] Cacciante L, Pietà Camilla Della, Rutkowski S, Cieślik B, Szczepańska-Gieracha J, Agostini M, Kiper P (2022). Cognitive telerehabilitation in neurological patients: systematic review and meta-analysis. Neurol Sci.

[15] Suso-Martí Luis, La Touche R, Herranz-Gómez Aida, Angulo-Díaz-Parreño Santiago, Paris-Alemany A, Cuenca-Martínez Ferran (2021). Effectiveness of telerehabilitation in physical therapist practice: an umbrella and mapping review with meta-meta-analysis. Phys Ther.

[16] Cottrell MA, Galea OA, O'Leary SP, Hill AJ, Russell TG (2017). Real-time telerehabilitation for the treatment of musculoskeletal conditions is effective and comparable to standard practice: a systematic review and meta-analysis. Clin Rehabil.

[17] Eccleston C, Blyth FM, Dear BF, Fisher EA, Keefe FJ, Lynch ME, Palermo TM, Reid MC, Williams ACDC (2020). Managing patients with chronic pain during the COVID-19 outbreak: considerations for the rapid introduction of remotely supported (eHealth) pain management services. Pain.

[18] Hernando-Garijo Ignacio, Ceballos-Laita Luis, Mingo-Gómez María Teresa, Medrano-de-la-Fuente Ricardo, Estébanez-de-Miguel Elena, Martínez-Pérez María Natividad, Jiménez-Del-Barrio Sandra (2021). Immediate effects of a telerehabilitation program based on aerobic exercise in women with fibromyalgia. Int J Environ Res Public Health.

[19] Lee J, Park S, Ju JH, Cho JH (2019). Application of a real-time pain monitoring system in Korean fibromyalgia patients: a pilot study. Int J Rheum Dis.

[20] Molinari G, García-Palacios Azucena, Roca P, Fernández-Llanio Comella Nagore, Botella C, Enrique (2018). The power of visualization: back to the future for pain management in fibromyalgia syndrome. Pain Med.

[21] Yuan Susan Lee King, Couto Letícia Assis, Marques Amélia Pasqual (2021). Effects of a six-week mobile app versus paper book intervention on quality of life, symptoms, and self-care in patients with fibromyalgia: a randomized parallel trial. Braz J Phys Ther.

[22] Bernardy K, Klose P, Welsch P, Häuser W (2019). Efficacy, acceptability and safety of Internet-delivered psychological therapies for fibromyalgia syndrome: a systematic review and meta-analysis of randomized controlled trials. Eur J Pain.

[23] Higgins JPT, Altman DG, Gøtzsche Peter C, Jüni Peter, Moher D, Oxman AD, Savovic J, Schulz KF, Weeks L, Sterne JAC, Cochrane Bias Methods Group, Cochrane Statistical Methods Group (2011). The Cochrane Collaboration's tool for assessing risk of bias in randomised trials. BMJ.

[24] Higgins JPT, Thompson SG, Deeks JJ, Altman DG (2003). Measuring inconsistency in meta-analyses. BMJ.

[25] Irwig L, Macaskill P, Berry G, Glasziou P (1998). Bias in meta-analysis detected by a simple, graphical test. graphical test is itself biased. BMJ.

[26] Egger M, Davey Smith G, Schneider M, Minder C (1997). Bias in meta-analysis detected by a simple, graphical test. BMJ.

[27] Ang DC, Chakr R, Mazzuca S, France CR, Steiner J, Stump T (2010). Cognitive-behavioral therapy attenuates nociceptive responding in patients with fibromyalgia: a pilot study. Arthritis Care Res (Hoboken).

[28] Williams David A, Kuper David, Segar Michelle, Mohan Niveditha, Sheth Manish, Clauw Daniel J (2010). Internet-enhanced management of fibromyalgia: a randomized controlled trial. Pain.

[29] Menga G, Ing S, Khan O, Dupre B, Dornelles AC, Alarakhia A, Davis W, Zakem J, Webb-Detiege T, Scopelitis E, Quinet R (2014). Fibromyalgia: can online cognitive behavioral therapy help?. Ochsner J.

[30] Vallejo Miguel A, Ortega José, Rivera Javier, Comeche María I, Vallejo-Slocker Laura (2015). Internet versus face-to-face group cognitive-behavioral therapy for fibromyalgia: a randomized control trial. J Psychiatr Res.

[31] Friesen LN, Hadjistavropoulos HD, Schneider LH, Alberts NM, Titov N, Dear BF (2017). Examination of an internet-delivered cognitive behavioural pain management course for adults with fibromyalgia: a randomized controlled trial. Pain.

[32] Hedman-Lagerlöf Maria, Hedman-Lagerlöf Erik, Axelsson Erland, Ljótsson Brjánn, Engelbrektsson Johanna, Hultkrantz Sofia, Lundbäck Karolina, Björkander Daniel, Wicksell Rikard K, Flink Ida, Andersson Erik (2018). Internet-delivered exposure therapy for fibromyalgia: a randomized controlled trial. Clin J Pain.

[33] Simister Heather D, Tkachuk Gregg A, Shay Barbara L, Vincent Norah, Pear Joseph J, Skrabek Ryan Q (2018). Randomized controlled trial of online acceptance and commitment therapy for fibromyalgia. J Pain.

[34] García-Perea Eva, Pedraz-Marcos Azucena, Martínez-Rodríguez Sandra Helena, Otones-Reyes Pedro, Palmar-Santos Ana Maria (2022). Effectiveness of a fibromyalgia online nursing consultation in the quality of life: a randomized controlled trial. Pain Manag Nurs.

[35] Serrat M, Coll-Omaña Mireia, Albajes K, Solé Sílvia, Almirall M, Luciano JV, Feliu-Soler A (2021). Efficacy of the FIBROWALK multicomponent program moved to a virtual setting for patients with fibromyalgia during the COVID-19 pandemic: a proof-of-concept RCT performed alongside the state of alarm in Spain. Int J Environ Res Public Health.

[36] Serrat M, Albajes K, Navarrete J, Almirall M, Lluch Girbés Enrique, Neblett R, Luciano JV, Moix J, Feliu-Soler A (2022). Effectiveness of two video-based multicomponent treatments for fibromyalgia: The added value of cognitive restructuring and mindfulness in a three-arm randomised controlled trial. Behav Res Ther.

[37] MoodGYM.

[38] Chen T, Or CK, Chen J (2021). Effects of technology-supported exercise programs on the knee pain, physical function, and quality of life of individuals with knee osteoarthritis and/or chronic knee pain: a systematic review and meta-analysis of randomized controlled trials. J Am Med Inform Assoc.

[39] Chen M, Wu T, Lv M, Chen C, Fang Z, Zeng Z, Qian J, Jiang S, Chen W, Zhang J (2021). Efficacy of mobile health in patients with low back pain: systematic review and meta-analysis of randomized controlled trials. JMIR mHealth uHealth.

[40] Tsang MP, Man GCW, Xin H, Chong YC, Ong MT, Yung PS (2022). The effectiveness of telerehabilitation in patients after total knee replacement: A systematic review and meta-analysis of randomized controlled trials. J Telemed Telecare.

[41] Rennie K, Taylor C, Corriero AC, Chong C, Sewell E, Hadley J, Ardani S (2022). The current accuracy, cost-effectiveness, and uses of musculoskeletal telehealth and telerehabilitation services. Curr Sports Med Rep.

[42] Winters JM (2002). Telerehabilitation research: emerging opportunities. Annu Rev Biomed Eng.

[43] Aragaki D, Luo J, Weiner E, Zhang G, Darvish B (2021). Cardiopulmonary telerehabilitation. Phys Med Rehabil Clin N Am.

[44] Häuser Winfried, Ablin J, Perrot S, Fitzcharles M (2017). Management of fibromyalgia: practical guides from recent evidence-based guidelines. Pol Arch Intern Med.

[45] Redfern J (2019). Can older adults benefit from smart devices, wearables, and other digital health options to enhance cardiac rehabilitation?. Clin Geriatr Med.

[46] Howard IM, Kaufman MS (2018). Telehealth applications for outpatients with neuromuscular or musculoskeletal disorders. Muscle Nerve.

