# Supplementary material for: The Efficacy and Safety of Telerehabilitation for Fibromyalgia: Systematic Review and Meta-analysis of Randomized Controlled Trials
Source: J Med Internet Res. 2023 Apr 25;25:e42090. doi: 10.2196/42090 (PMC10170363; doi:10.2196/42090)
Supplement: Multimedia Appendix 1 [file jmir_v25i1e42090_app1.docx]

**Multimedia Appendix 1:** Search Strategy

**PubMed:**

#1: "Telerehabilitation"[Mesh]

#2: "Telemedicine"[Mesh]

#4: "Videoconferencing"[Mesh]

#5: "Wireless Technology"[Mesh]

#6: "Computer Communication Networks"[Mesh]

#**7: (Telerehabilitation OR telemedicine OR Mobile health OR Remote Rehabilitation OR Internet-Based Intervention OR mHealth OR mobile applications OR smartphone** OR Videoconferencing OR Wireless Technology OR Computer Communication Networks) [All Fields]

#8: 1 OR 2 OR3 OR 4 OR 5 OR 6 OR 7

#9: "Fibromyalgia"[Mesh]

#10: (fibromyalgia OR fibromyalgia syndrome OR fibromyalgia-fibromyositis syndrome) [All Fields]

#11: #9 OR #10

#12: #8 AND #11

**Web of Science:**

#**1:TS=(Telerehabilitation) OR (telemedicine)) OR (Mobile health) OR (Remote Rehabilitation) OR (Internet-Based Intervention) OR (mHealth) OR (mobile applications) OR (smartphone)** OR (Videoconferencing) OR (Wireless Technology) OR (Computer Communication Networks)

#2: TS=(fibromyalgia) OR (fibromyalgia syndrome) OR (fibromyalgia-fibromyositis syndrome)

#3: #1AND #2

**ScienceDirect**

(Telerehabilitation OR telemedicine OR Internet-Based Intervention OR eHealth OR mobile applications) AND (fibromyalgia OR fibromyalgia syndrome OR fibromyalgia-fibromyositis syndrome)

**Ovid MEDLINE**

#1: Telemedicine/ or Telerehabilitation

#2: Internet/ or Internet-Based Intervention

#3: Mobile Applications

#4: (Telerehabilitation or telemedicine or Mobile health or Remote Rehabilitation or Internet-Based Intervention or eHealth or mobile applications or smartphone).mp. [mp=title, abstract, original title, name of substance word, subject heading word, floating sub-heading word, keyword heading word, organism supplementary concept word, protocol supplementary concept word, rare disease supplementary concept word, unique identifier, synonyms]

#5: 1 or 2 or 3 or 4

#6: Fibromyalgia

#7: (fibromyalgia or fibromyalgia syndrome or fibromyalgia-fibromyositis syndrome).mp. [mp=title, abstract, original title, name of substance word, subject heading word, floating sub-heading word, keyword heading word, organism supplementary concept word, protocol supplementary concept word, rare disease supplementary concept word, unique identifier, synonyms]

#8: #6 or #7

#9: #5 and #8

**Cochrane Library**

#1: Telerehabilitation OR telemedicine OR Internet-Based Intervention OR eHealth OR mobile applications OR Videoconferencing OR Wireless Technology OR Computer Communication Networks

#2: fibromyalgia OR fibromyalgia syndrome OR fibromyalgia-fibromyositis syndrome

#3: Randomized Controlled Trial OR Double-Blind Method OR Single-Blind Method OR Clinical Trial OR Controlled Clinical Trial

#4: #1 AND #2 AND #3

**EMBASE**

#1: 'telerehabilitation'/exp OR 'telemedicine'/exp OR 'internet-based intervention'/exp OR 'ehealth'/exp OR 'mobile applications'/exp OR 'videoconferencing'/exp OR 'wireless technology'/exp OR 'computer communication networks'/exp

#2: telemetry:ti,ab,kw OR telerehabilitation:ti,ab,kw OR telemedicine:ti,ab,kw OR ehealth:ti,ab,kw OR 'mobile health':ti,ab,kw OR 'remote rehabilitation':ti,ab,kw OR mhealth:ti,ab,kw OR 'mobile applications':ti,ab,kw OR smartphone:ti,ab,kw OR technolog*:ti,ab,kw

#3: #1 OR #2

#4: 'fibromyalgia'/exp

#5: 'myofascial pain syndrome, diffuse':ti,ab,kw OR fibromyalgias:ti,ab,kw OR 'fibromyalgia-fibromyositis syndrome':ti,ab,kw OR 'fibromyalgia syndrome':ti,ab,kw

#6: #4 OR #5

#7: #6 OR #3

PEDro

1: "Telerehabilitation" AND "Fibromyalgia"

2: " Telemedicine " AND "Fibromyalgia"

3: " Videoconferencing " AND "Fibromyalgia"

4: " Wireless Technology " AND "Fibromyalgia"

5: "Computer Communication Networks " AND "Fibromyalgia"

6: " Mobile health " AND "Fibromyalgia"

7: " **mHealth** " AND "Fibromyalgia"
