# Supplementary material for: The Efficacy and Safety of Telerehabilitation for Fibromyalgia: Systematic Review and Meta-analysis of Randomized Controlled Trials
Source: J Med Internet Res. 2023 Apr 25;25:e42090. doi: 10.2196/42090 (PMC10170363; doi:10.2196/42090)
Supplement: Multimedia Appendix 2 [file jmir_v25i1e42090_app2.docx]

**Multimedia Appendix 2**

**Funnel plots**


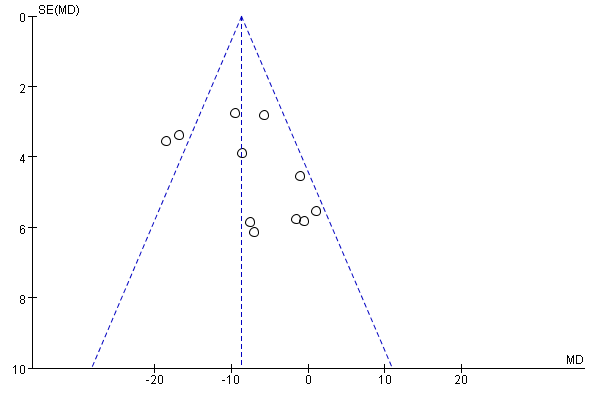
Figure 1: Funnel plot of studies included in meta-analysis assessing FIQ scale.

FIQ: fibromyalgia impact questionnaire; MD: mean difference; SE: standard Error.


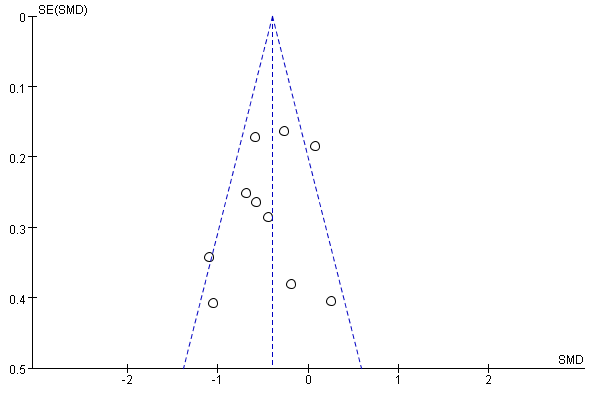
Figure 2: Funnel plot of studies included in meta-analysis assessing depression

MD: mean difference; SE: standard Error.
